# Difficult Cases

**Published:** 1888-08-04

**Authors:** 


### Difficult Cases.

[Replies endorsed "Difficult Cases," should be addressed to the Editor, The Lodge, Porchester Square, W.]

### A HOME WANTED.

Could any of your readers assist me in finding a place as needle-woman or sewing-maid for a young woman of 24, who has had one leg amputated? She is now in good health, but must go where the work is light. She is good at her needle, and can be well recommended in every way. She has been in service before. E. B. Carr-Gomm.

### A MELANCHOLY CASE.

A lady writes: "Some friends of mine are in great trouble, as the wife and mother has gone out of her mind. She is better, but not nearly well, and has taken such a dislike to some members of her family, who are all devoted to her. Can you tell me any nice place where she would be happy for two or three months? She is a little over 50, and this came on quite suddenly, after an attack of rheumatic gout, so it is possible she may quite recover after a time. Her husband is a clergyman, and they are poor, so cannot afford very much. I know you will suggest some suitable place, if you can, so I won't apologise for troubling you.

[We should advise our correspondent to recommend this case being placed under the care of Mr. Prichard, Abington Abbey Retreat, Northampton, which is in every sense a home for the mentally afflicted.—Ed. *T. H.*]

### A NURSE'S APPEAL FOR HER PATIENT.

Being a regular subscriber to The Hospital, I am writing to ask you to insert in its columns a query for information or advice as to this sad case: A young girl, aged 18, formerly an enamel worker, has been deprived entirely of her eyesight through lead poisoning and meningitis, so that any recovery is hopeless. Her family are very poor, and have been out of work, so that on her leaving this hospital (where she has been kept four months) she has little or no means of supporting herself. Is there any free blind institution? or one where by payment of a small sum she could be put into the way of earning a little basket work, etc.? She has a good voice, if it could be turned to account. She is very fond of children, and is a nice girl, fairly strong. I should be so much obliged if you could aid me in this matter through your paper. I am leaving this hospital next week, and the girl, too, will have to leave almost directly. I am very anxious for her not to have to go adrift—hopeless, penniless, and blind at her age. She does not seem to take to knitting, but is a sharp girl, and could easily pick up some work if rightly started. She is also pleasant, good tempered, trustful, and honest.

Nurse Mary.

